# Insights into Structural, Electronic, and Transport Properties of Pentagonal PdSe_2_ Nanotubes Using First-Principles Calculations

**DOI:** 10.3390/nano13111728

**Published:** 2023-05-25

**Authors:** Nguyen Thanh Tien, Pham Thi Bich Thao, Nguyen Hai Dang, Nguyen Duy Khanh, Vo Khuong Dien

**Affiliations:** 1 College of Natural Sciences, Can Tho University, Can Tho 90000, Vietnam; ptbthao@ctu.edu.vn (P.T.B.T.); nhdang@nctu.edu.vn (N.H.D.); 2 Faculty of Fundamental Science, Nam Can Tho University, Can Tho 90000, Vietnam; 3 High-Performance Computing Laboratory (HPC Lab), Information Technology Center, Thu Dau Mot University, Thu Dau Mot 75100, Vietnam; khanhnd@tdmu.edu.vn; 4 Department of Physics, National Cheng Kung University, Tainan 701, Taiwan; vokhuongdien@gmail.com

**Keywords:** nanotube, pentagon, palladium diselenide (PdSe_2_), structural properties, electronic properties, transport properties, strain, first-principles calculations, electronic devices, electromechanical sensors

## Abstract

One-dimensional (1D) novel pentagonal materials have gained significant attention as a new class of materials with unique properties that could influence future technologies. In this report, we studied the structural, electronic, and transport properties of 1D pentagonal PdSe_2_ nanotubes (p-PdSe_2_ NTs). The stability and electronic properties of p-PdSe_2_ NTs with different tube sizes and under uniaxial strain were investigated using density functional theory (DFT). The studied structures showed an indirect-to-direct bandgap transition with slight variation in the bandgap as the tube diameter increased. Specifically, (5 × 5) p-PdSe_2_ NT, (6 × 6) p-PdSe_2_ NT, (7 × 7) p-PdSe_2_ NT, and (8 × 8) p-PdSe_2_ NT are indirect bandgap semiconductors, while (9 × 9) p-PdSe_2_ NT exhibits a direct bandgap. In addition, under low uniaxial strain, the surveyed structures were stable and maintained the pentagonal ring structure. The structures were fragmented under tensile strain of 24%, and compression of −18% for sample (5 × 5) and −20% for sample (9 × 9). The electronic band structure and bandgap were strongly affected by uniaxial strain. The evolution of the bandgap vs. the strain was linear. The bandgap of p-PdSe_2_ NT experienced an indirect–direct–indirect or a direct–indirect–direct transition when axial strain was applied. A deformability effect in the current modulation was observed when the bias voltage ranged from about 1.4 to 2.0 V or from −1.2 to −2.0 V. Calculation of the field effect I–V characteristic showed that the on/off ratio was large with bias potentials from 1.5 to 2.0 V. This ratio increased when the inside of the nanotube contained a dielectric. The results of this investigation provide a better understanding of p-PdSe_2_ NTs, and open up potential applications in next-generation electronic devices and electromechanical sensors.

## 1. Introduction

The discovery of novel 1D structures has an important role in the development of new optoelectronic devices [1,2,3]. This becomes more important with materials that have a pentagonal structure [4]. Due to the unique geometry of these materials, they exhibit many interesting physico-chemical properties, and promise to have many other broad applications such as in batteries, catalysis, and thermoelectricity [5,6,7,8,9]. Nanostructured 1D materials have been synthesized or designed with structures such as nanowires [10,11], nanoribbons [12,13,14], and nanotubes [15,16]. One-dimensional nanostructures often have many advantages in the development of next-generation devices because of the satisfaction of the device miniaturization [17,18]. Many single- and multi-walled nanotube structures based on hexagonal transition metal dichalcogenides (TMD) have been synthesized. Their electronic properties and optical spectra have been studied [19,20,21]. The research results recognize many new properties compared to the parent 2D structure because of structural symmetry and van der Waals interactions between the layers.

Very recently, scientists working at the Center for Nanophase Materials Sciences, Oak Ridge National Laboratory, United States, reported that a pentagonal PdSe_2_ nanoribbon (p-PdSe_2_ NR) was fabricated through a combined top-down and bottom-up approach. This structure is confirmed to be stable in air [22]. They also investigated the electronic properties and confirmed that the p-PdSe_2_ NR has metallic properties. This work also studied the electron transport properties by setting up a two-terminal field-effect transistor. The I–V characteristic measurement results confirm the metallic properties of the p-PdSe_2_ NR. However, they did not show the edge configuration of the studied structure. By means of DFT calculation, we have systematically studied the electronic features and electron transport of p-PdSe_2_ NR structures. We have shown that the electronic properties of p-PdSe_2_ NRs strongly depend on the structure edge [23]. With nanoribbons, the electron transport property can be controlled via the edge design of the nanoribbon structure. However, compared with nanoribbons, nanotubes usually have better mechanical properties and are more stable [24,25,26]. Carbon nanotubes have been successfully integrated with other elements to create optoelectronic devices such as LEDs, field-effect transistors, and detectors [27,28] Therefore, it is necessary to study the electronic properties and electron transport of p-PdSe_2_ NTs systematically. Understanding their key properties will help to apply them in the development of novel devices.

In this work, we studied the structural properties, electronic properties, and electron transport of p-PdSe_2_ single-walled NTs with various diameters using theoretical calculations based on the first principle. With a 1D structure, the electronic properties can be controlled via deformation along the structure axis; this study also investigated this effect. A field-effect transistor model with small-sized p-PdSe_2_ NTs was also considered. Both dielectric-core and air-core field-effect transistors were designed and dissected.

## 2. Computational Details

Structural relaxation and electronic band structure calculations were carried out using DFT with the GGA-PBE exchange–correlation function [29] and double-zeta polarized basis sets [30], implemented in the Atomistix ToolKit (ATK) software package [31]. We used a *k*-point grid of (1 × 1 × 21) with an energy cut-off of 680 eV for calculation. To avoid interactions between periodic images, a vacuum of 15 Å thickness was applied in the x and y directions. The convergence precision of the energy for the maximum residual force on each atom was 0.01 eV/Å. The Fermi level (*E*_F_) was set to zero in all calculations.

We also used ATK for the simulation of the device. The studied device model has a two-probe geometry. It consists of a left electrode, a central region, and a right electrode. The three regions have the same boundary conditions (BCs) in the two lateral directions perpendicular to the left–right electron transport direction. The left and right electrodes were assumed to have bulk properties, and the first step of the device simulation was to perform a bulk calculation of each electrode with periodic BCs in the transport direction. Then, using Bloch’s theorem, we described the wave functions in terms of transverse *k*-points, and to seamlessly connect the three regions, the same *k*-point sampling was used in the transverse directions for all three regions. A very dense *k*-point grid of (1 × 1 × 200) electrodes was established in the transport direction. To reduce the computational effort of DFT, the tight-binding ATK-SE tool (semi-empirical model simulation) was used in the calculation of the device [32].

The left and right electrodes were modeled in their ground states with chemical potentials $\mu_{L}$ and $\mu_{R}$, respectively. The electronic structures of the isolated electrodes were defined with respect to an arbitrary energy reference.

(1) $$\mu_{L}-\mu_{R}=-eV_{bias},$$

where $V_{bias}$ is the bias voltage applied on the electrodes. The non-equilibrium Green function (NEGF) approach was used to calculate the non-equilibrium electron density. Once the self-consistent non-equilibrium density matrix was obtained, it was possible to calculate the transmission. The transmission coefficient *T(E)* at the electron energy *E* was obtained using the retarded Green function [33], as follows:

(2) $$T(E)=\mathrm{Tr}\left[G(E)\gamma^{L}(E)G^{†}(E)\gamma^{R}(E)\right],$$

and the electrical current was calculated using the Landauer formula,

(3) $$I=\frac{2e}{h}\int_{-\infty }^{\infty }T(E)\left[f\left(\frac{E-\mu_{L}}{k_{B}T_{L}}\right)-f\left(\frac{E-\mu_{R}}{k_{B}T_{R}}\right)\right]dE.$$

where f(L/R) is the Fermi distribution function of the electronic carriers in the leads.

## 3. Results

### 3.1. Effect of Size on the Structural and Electronic Properties of Pentagonal Palladium Diselenide Nanotubes

#### 3.1.1. Structure and Energetic Stability

**Figure 1:**
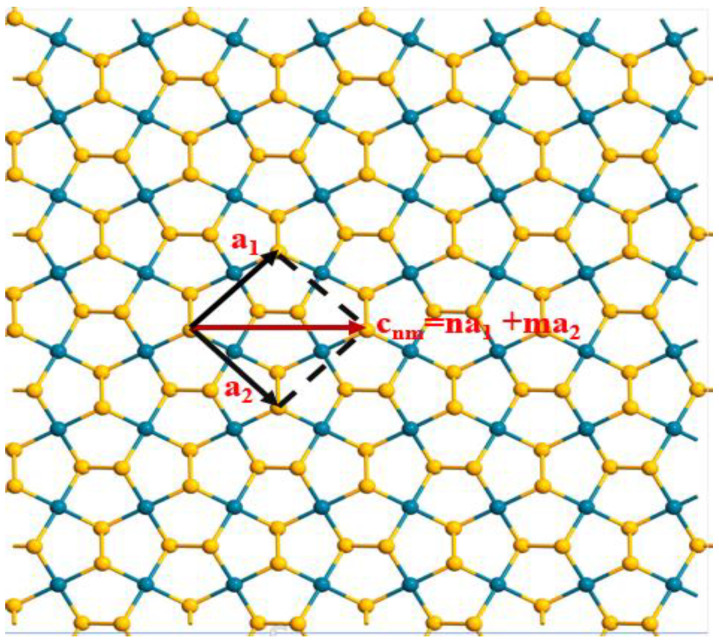
Illustration of the chiral vector of p-PdSe_2_ NTs.

**Figure 2:**
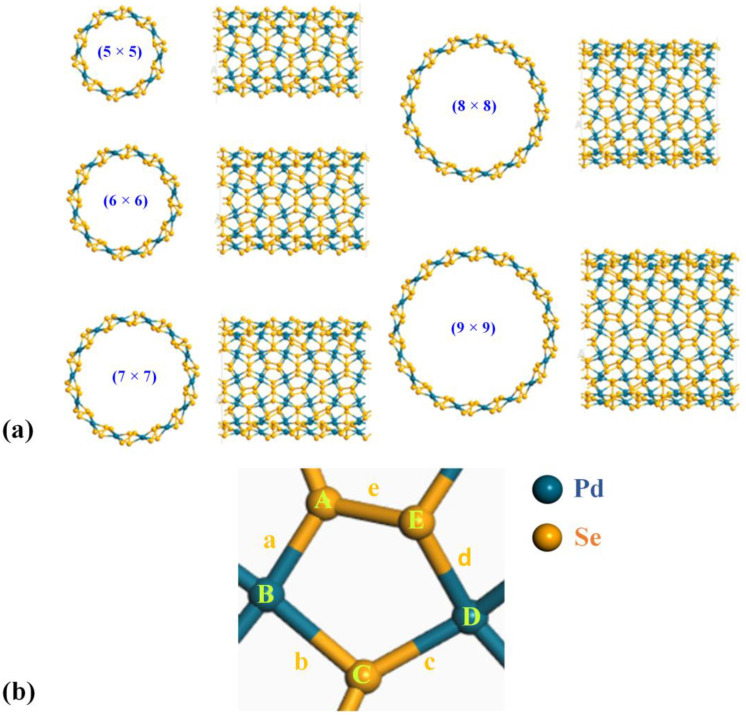
(**a**) Illustration of p-PdSe_2_ NT structures of different diameters: (5 × 5), (6 × 6), (7 × 7), (8 × 8), (9 × 9). (**b**) Enlarged pentagonal ring in the p-PdSe_2_ NT structure. The blue and yellow balls represent Pd and Se atoms, respectively. The Pd–Se bond lengths are denoted by a, b, c, and d; the Se–Se bond length is denoted by e. The bond angles are denoted by A, B, C, D, and E.

The geometry of a nanotube is determined by the chiral indices in the **a_1_** and **a_2_** in-plane directions, which are represented by *n* and *m* (where *n* and *m* are positive integers) [34]. In this study, we considered chiral vectors *n* = *m*. As depicted in Figure 1, starting from 2D pentagonal PdSe_2_, (*n* × *n*) p-PdSe_2_ NTs can be constructed by rolling up the sheet along the chiral vectors. The larger the chiral indices, the larger the nanotube diameter, and the less curved the nanotube. With small-diameter pentagonal nanotubes, a curved effect appears, and the nanotubes are less stable [35]. In contrast, the large-diameter nanotubes exhibit properties of the 2D parent material. In this study, we mainly studied medium-diameter (*n* × *n*) nanotubes that exhibit stability. The structures implemented in this research were (5 × 5) p-PdSe_2_ NT, (6 × 6) p-PdSe_2_ NT, (7 × 7) p-PdSe_2_ NT, (8 × 8) p-PdSe_2_ NT, and (9 × 9) p-PdSe_2_ NT, as shown in Figure 2.

To evaluate the stability of the optimized structures, the cohesive energy *E_c_* is defined as follows:

(4) $$E_{c}=\frac{E_{total}-n_{\mathrm{Pd}}E_{\mathrm{Pd}}-n_{\mathrm{Se}}E_{\mathrm{Se}}}{n_{\mathrm{Pd}}+n_{\mathrm{Se}}},$$

where *E_total_* is the optimized total energy of p-PdSe_2_ NTs; *E*_Pd_ and *E*_Se_ are the energies of isolated Pd and Se atoms, respectively; *n*_Pd_ and *n*_Se_ indicate the number of Pd and Se atoms in the p-PdSe_2_ NTs. As presented in Table 1, the cohesive energy of all investigated p-PdSe_2_ NTs varied between −1.995 and −2.020 eV/atom, indicating that these optimized p-PdSe_2_ NTs are energetically stable. Among them, the (9 × 9) p-PdSe_2_ NT structure is the most stable due to it having the lowest cohesive energy. Specifically, the cohesive energy decreased slightly when the tube diameter increased; this result is similar to that in a previous study by Kulin et al. [36].

The calculated cohesive energy of the 2D p-PdSe_2_ system in the supercell model was −4.030 eV/atom. This value is consistent with that in a previous study by Dhara Raval et al. (−4.300 eV/atom) [37]. It seems that the cohesive energy of the large-diameter p-PdSe_2_ nanotubes is asymptotically close to the cohesive energy of the 2D p-PdSe_2_ system.

Table 2 presents the bond lengths and bond angles of a central pentagonal ring of p-PdSe_2_ NTs with different diameters. In general, the bond lengths of the various p-PdSe_2_ NTs remained almost unchanged. The Pd–Se bond length ranged from 2.49 Å to 2.52 Å, while the Se–Se bond length was approximately 2.46 Å. These values are similar to the Pd–Se (2.48 Å) and Se–Se (2.43 Å) bond lengths in the 2D p-PdSe_2_ structure [36,37,38]. The obtained results are also similar to those in a previous study on penta-graphene nanotubes (PGNTs) by Wang et al.; the bond length does not change significantly when the tube diameter changes [39]. However, when forming nanotubes, the bond angles change. The two bond angles involved in the Se–Se bond change the most. This is because the Se–Se bond is a covalent bond, while the Pd–Se bond is an ionic bond [40,41].

#### 3.1.2. Electronic Properties

**Figure 3:**
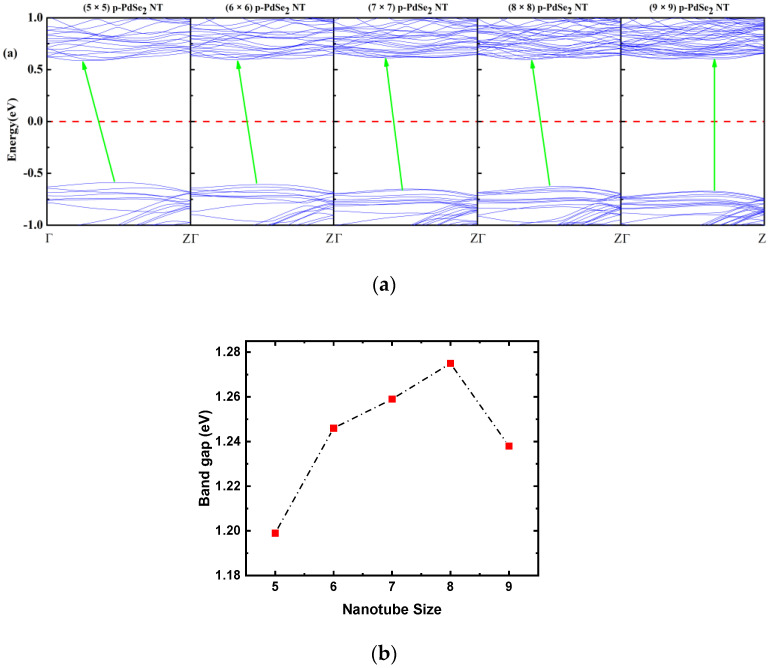
(**a**) Electronic band structures of the (5 × 5), (6 × 6), (7 × 7), (8 × 8), and (9 × 9) p-PdSe_2_ NTs. The green arrow line is the line connecting the valence band maximum and the conduction band minimum. (**b**) The bandgap value vs. nanotube size.

The band structures of p-PdSe_2_ NTs with different diameters are depicted in Figure 3. The calculation results show that the bandgaps (Eg) of (5 × 5) p-PdSe_2_ NT, (6 × 6) p-PdSe_2_ NT, (7 × 7) p-PdSe_2_ NT, (8 × 8) p-PdSe_2_ NT, and (9 × 9) p-PdSe_2_ NT were 1.199 eV, 1.246 eV, 1.259 eV, 1.275 eV, and 1.238 eV, respectively. This shows that the Eg of p-PdSe_2_ NTs with different diameters is slightly different. The bandgap of the NTs is asymptotic and smaller than that of 2D p-PdSe_2_ (Eg ~1.38 eV under the GGA approach, and ~2.0 eV under the GW-BSE approach) [35,41,42]. Interestingly, (5 × 5) p-PdSe_2_ NT, (6 × 6) p-PdSe_2_ NT, (7 × 7) p-PdSe_2_ NT, and (8 × 8) p-PdSe_2_ NT are indirect bandgap semiconductors, whereas (9 × 9) p-PdSe_2_ NT has a direct bandgap. The indirect bandgap of (5 × 5) p-PdSe_2_ NT switched to the direct bandgap of (9 × 9) p-PdSe_2_ NT [36]. In addition, a study of PGNTs also showed that the bandgap of PGNTs varied slightly when the diameter of the PGNTs was changed [39]. Thus, NTs of different sizes formed from a 2D pentagonal material have insignificant differences in bandgap, and an indirect-to-direct bandgap transition occurs. We suggest that this is a remarkable feature that can influence the electronic, transport, and optical properties of NTs with different diameters formed from a 2D pentagonal material.

### 3.2. Effect of Strain on the Structural and Electronic Properties of p-PdSe_2_ Nanotubes

#### 3.2.1. Structure and Energetic Stability

**Figure 4:**
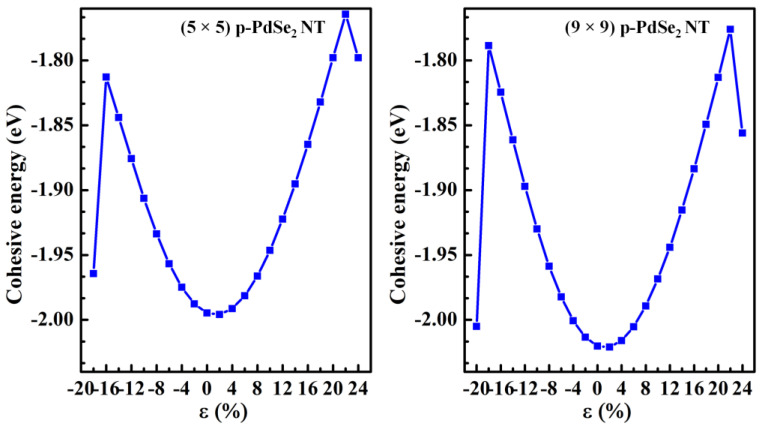
The cohesive energy of p-PdSe_2_ NTs under uniaxial strain in the z direction.

In this section, we investigate the effect of uniaxial strain along the Oz direction of the (5 × 5) p-PdSe_2_ and (9 × 9) p-PdSe_2_ NTs. For (5 × 5) p-PdSe_2_ NT, the compressive and tensile strains were ε = −16%, −14%, −12%, −10%, −8%, −6%, −4%, −2%, 0%, 2%, 4%, 6%, 8%, 10%, 12%, 14%, 16%, 18%, 20%, and 22%. For (9 × 9) p-PdSe_2_ NT, ε = −18%, −16%, −14%, −12%, −10%, −8%, −6%, −4%, −2%, 0%, 2%, 4%, 6%, 8%, 10%, 12%, 14%, 16%, 18%, 20%, and 22%. These structures were optimized and their electronic characteristics were investigated using the same parameters in Section 2. The strain was calculated using the formula

(5) $$\varepsilon =\frac{c-c_{0}}{c_{0}},$$

where $c_{0}$ and c are the lattice constants in the Oz direction of the pristine and strained structures. The minus and plus signs are uniaxial compressive strain and tensile strain, respectively. The cohesive energies of (5 × 5) p-PdSe_2_ NT and (9 × 9) p-PdSe_2_ NT under uniaxial strain are shown in Figure 4. The cohesive energy of (5 × 5) p-PdSe_2_ NT without strain was −1.9946 eV, while this value for (9 × 9) p-PdSe_2_ NT was −2.0202 eV. For both (5 × 5) p-PdSe_2_ NT and (9 × 9) p-PdSe_2_ NT, the cohesive energy dependence on uniaxial strain was parabolic. The cohesive energy of the two uniaxially strained structures changed significantly and showed a negative value. Both studied structures were stable up to 22% tensile strain. Meanwhile, the (5 × 5) p-PdSe_2_ and (9 × 9) p-PdSe_2_ NTs had a broken pentagonal ring structure when they were subjected to −18% and −20% compressive strain, respectively. Obviously, nanotubes with larger diameters are more compressible.

**Figure 5:**
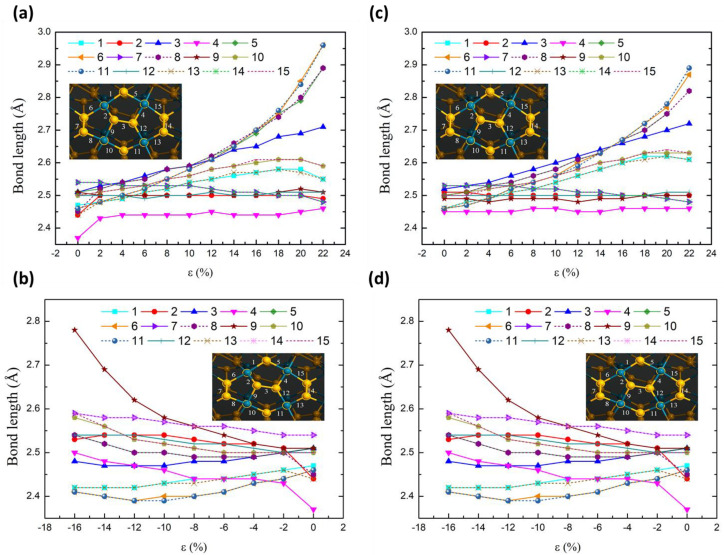
The bond lengths vs. strain of (5 × 5) p-PdSe_2_ NT ((**a**) tensile and (**b**) compressive strain) and (9 × 9) p-PdSe_2_ NT ((**c**) tensile and (**d**) compressive strain) in the four chosen pentagonal rings.

To investigate the structural change caused by strain, the bond lengths in the four pentagonal rings were measured in terms of the strain step (Figure 5). Generally, the bond length increases with increasing tensile strain and decreases with increasing compressive strain. For tensile strain, the bond lengths around the large hexagonal ring (made up of four small pentagons) recorded a significant change compared to the internal bonds. For the compressive strain, the bond lengths inside the large hexagonal ring recorded a significant change compared to the outer bonds. Symmetric bond pairs such as 1-13, 5-8, 6-11, 7-14, and 10-15 showed the same bond length variation for both tensile and compressive strain. Symmetric bond pairs such as 9-4 and 2-12 showed opposite variations for both tensile and compressive strain. This change involves the interaction of the Se–Se and Se–Pd bonds by applying external force along the tube’s axis. Bonds arranged in the axial direction of the tube change more, and vice versa, with less change in the direction perpendicular to the axis.

#### 3.2.2. Electronic Properties

The electronic band structures of the low-energy electronic states in the presence of strain (stretch and compression) considered here were calculated, as shown in Figure S1 for (5 × 5) p-PdSe_2_ NT and in Figure S2 for (9 × 9) p-PdSe_2_ NT.

**Figure 6:**
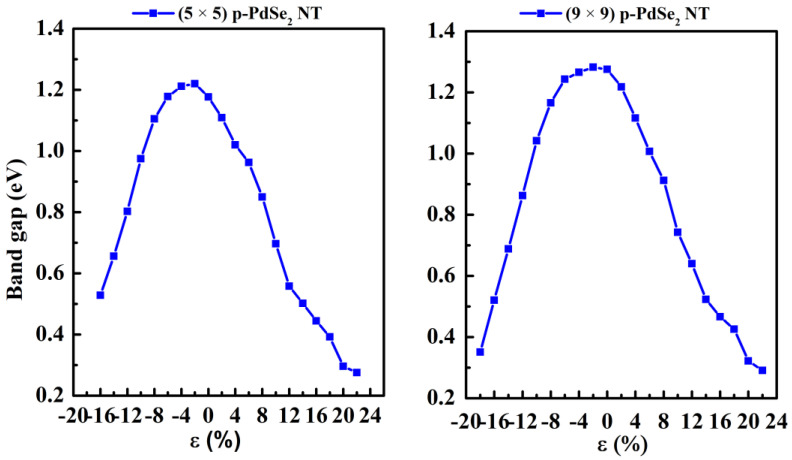
The bandgap of (5 × 5) p-PdSe_2_ NT and (9 × 9) p-PdSe_2_ NT vs. strain.

The calculation results show that the strained NTs are semiconductors. However, the conduction band minimum (CBM) and valence band maximum (VBM) varied considerably. This can lead to a bandgap transition (direct–indirect). The bandgap value (Figure 6) changed significantly when the sample was deformed. The bandgap exhibited a slightly linear trend in the case of stretching strain. On the other hand, for compressive strain, the bandgap behaved more intricately. Initially, it slightly increased or remained unchanged, but as the compression increased, it started to exhibit a linear trend in variation.

The bandgap varied with a linear trend in the case of compressive strain and a slightly linear trend in the case of stretching strain. This holds great potential for engineering tunable electronic devices through mechanical strain [43,44].

For (5 × 5) p-PdSe_2_ NT, the bandgap changed from indirect to direct in the case of the compressed NT. An indirect bandgap was retained in the case of the stretched NT. The CBM shifted to a high symmetry point Γ. For (9 × 9) p-PdSe_2_ NT, the bandgap changed from direct to indirect. In the case of stretching deformation, both the VBM and CBM tended to shift towards a high symmetry point Γ in the Brillouin region.

The electronic configuration of Pd is [Kr]4d^10^, and that of Se is [Ar]4s^2^3d^10^4p^4^. Due to the deformation, the densities of the d-orbital state of the Pd atom and the p-orbital state of the Se atom change. This leads to a change in the orbital space configuration and orbital hybridization. This is the cause of the transformation in the form of the bandgap. The change in the form of the bandgap caused by the strain effect is illustrated in Figure S3 (compressive strain) and Figure S4 (tensile strain) for the (5 × 5) p-PdSe_2_ NT case. This effect has also been observed experimentally in some strained 2D TMD [45].

### 3.3. Electron Transport Properties of Pentagonal PdSe_2_ Nanotubes

#### 3.3.1. Strain Effect

**Figure 7:**
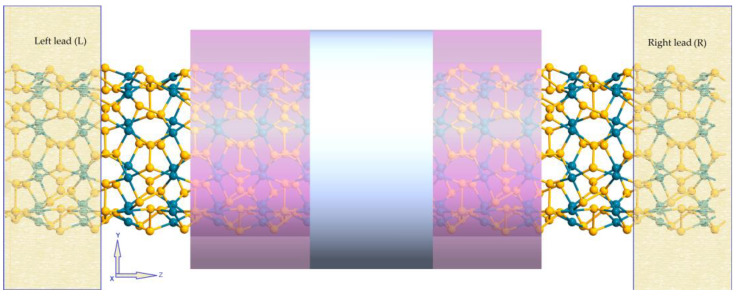
The two-probe device model based on p-PdSe_2_ nanotubes. The cylindrical rings represent dielectrics (lotus pink) and metals (silver).

Nanotubes are usually designed in the active region of the device [46,47]. To understand the basic electron transport properties of the device based on the studied materials, we modeled the two-probe device mentioned in Section 2. The device model is shown in Figure 7.

**Figure 8:**
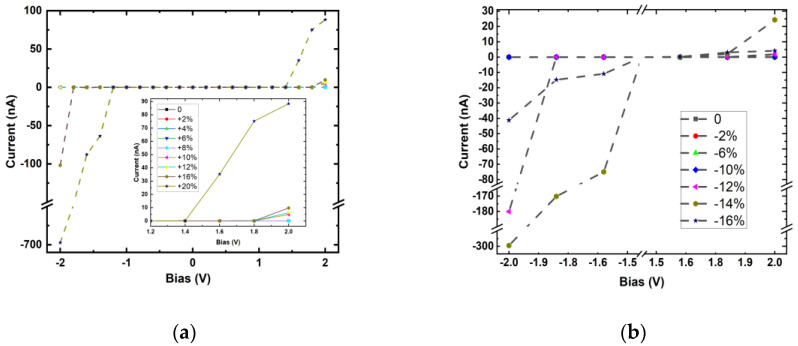
The I–V characteristics of (**a**) the stretched and (**b**) compressed (5 × 5) p-PdSe_2_ NTs. The inset in figure (**a**) is a magnified image of the applied voltage range of 1.2 V to 2.0 V.

As an example, we chose (5 × 5) p-PdSe_2_ NT to design the device. The device includes three regions: semi-infinite left lead (L), central scattering central region (CR), and semi-infinite right lead (R). Each lead consists of two unit cells, and the length of the scattering region in each device is three unit cells. First, the I–V characteristics were investigated in the strain situation without considering the field effect. Figure 8 shows the calculated I–V results of (5 × 5) p-PdSe_2_ NT in two cases of stretching and compressive strain along the nanotube axis.

The I–V calculation results confirm the semiconductor properties of the p-PdSe_2_ NT structures. The value of the current changed markedly in the case of stretching strain with a high bias voltage (2.0 V or −2.0 V). This represents a linear change in the electric current value with a bias voltage of about 2.0 V in the case of stretching strain. This effect was not observed for compressive strain when the compression was significant. This result shows that p-PdSe_2_ NTs can be used to develop a low-strain sensor.

#### 3.3.2. Field Effect

**Figure 9:**
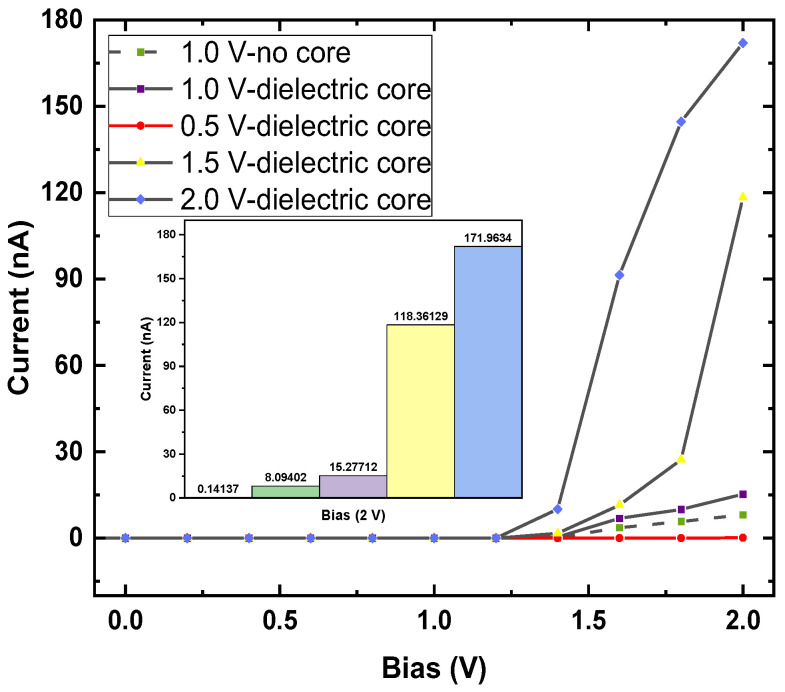
The I–V characteristics of the field-effect transistor model based on (5 × 5) p-PdSe_2_ NT. The inset in figure (a) is a magnified image of the applied voltage range of 1.2 V to 2.0 V. The inset figure shows the current values at the 2 V bias potential for the cases 0.5 V dielectric, 1.0 V no core, 1.0 V dielectric, 1.5 V dielectric, and 2.0 V dielectric.

A field-effect transistor (FET) was also modeled, consisting of a source (S), a drain (D), and a gate (G) terminal (Figure 7), where the current in the semiconducting channel between the S and D terminals is modulated by the electric field generated by the voltage of the G terminal and the voltage applied between the S and D terminals [48,49]. The gate potentials were applied at 0.5, 1.0, 1.5, and 2.0 V. The junction between the metal G terminal and the nanotube was inserted into a dielectric layer with a dielectric constant ∈ = 4. To evaluate the ballistic transport effect, this study investigated two situations with a dielectric core and no dielectric core inside the nanotubes (Figure S5b,c). The I–V characteristics of the p-PdSe_2_ NTs in the field-effect situation are shown in Figure 9.

The calculation results show that the on/off effect was very obvious when applying the 2.0 V voltage to the D–S. This effect was even more obvious when the inside of the p-PdSe_2_ nanotube had a dielectric core. The current values were 0.1414, 15.2771, 118.3613, and 171.9634 nA with 0.5, 1.0, 1.5, and 2.0 V gate voltages, respectively. The current value with zero bias was 9.419 × 10^−5^ nA. Therefore, the on/off ratios were 1.501, 162.184, 1.256.542, and 1.825.591, respectively. A high on/off ratio of ~10^7^ was achieved with a 2.0 V bias and 2.0 V gate voltage. This ratio is one order of magnitude smaller than previous studies for similar material systems [50,51] by Zhou et al.

## 4. Discussion

In the near future, relevant research may be extended regarding failure and molecular adsorption mechanisms to further validate the electronic properties and possible sensor applications.

## 5. Conclusions

In summary, this work systematically investigated the structural, electronic, and transport properties of p-PdSe_2_ NTs based on first-principles calculations combined with the non-equilibrium Green function method. The NT structures were stable and exhibited evident 1D electronic characteristics with tube sizes varying from (5 × 5) to (9 × 9). The (9 × 9) p-PdSe_2_ NT structure was the most stable, possessing the lowest cohesive energy of −2.020 eV. The electronic band structure changed from indirect to direct form as the tube size increased. The bandgap value changed with the deformation, similar to a parabolic curve. This study clearly illustrates the results of electronic characteristic modulation by uniaxial strain along the tube axis. The V–A curve of the nanotube-based bipolar device model also exhibited a modulation characteristic. The research results show that the calculated on/off ratio was significantly large. This ratio increased when a dielectric core was placed inside the tube. The research results systematically contribute to the development of electronic devices, e.g., electromechanical sensors and field-effect transistors, based on p-PdSe_2_ NTs.

## Figures and Tables

**Table 1:** The cohesive energy of the investigated (*n* × *n*) p-PdSe_2_ NTs.

(5 × 5)	(6 × 6)	(7 × 7)	(8 × 8)	(9 × 9)
−1.995 eV/atom	−2.006 eV/atom	−2.013 eV/atom	−2.017 eV/atom	−2.020 eV/atom

**Table 2:** The bond lengths and bond angles of a central pentagonal ring of (*n* × *n*) p-PdSe_2_ NTs.

(*n* × *n*)	a	b	c	d	e	A	B	C	D	E
(5 × 5)	2.51	2.50	2.51	2.50	2.46	92.72	98.72	111.88	77.32	104.74
(6 × 6)	2.50	2.50	2.51	2.50	2.46	93.60	98.35	112.06	78.59	103.21
(7 × 7)	2.50	2.50	2.51	2.50	2.46	94.23	98.05	112.25	79.43	102.16
(8 × 8)	2.49	2.50	2.52	2.50	2.46	94.70	97.96	112.33	80.14	101.56
(9 × 9)	2.49	2.50	2.52	2.50	2.46	95.23	97.86	112.34	80.84	100.98

The Pd–Se bond lengths (Å) are denoted by a, b, c, and d; the Se–Se bond length is denoted by e. The bond angles (degree) are denoted by A, B, C, D, and E (see Figure 2).

## Data Availability

Data are contained within the article.

## Supplementary Materials

The following supporting information can be downloaded at: https://www.mdpi.com/article/10.3390/nano13111728/s1, Figure S1: Electronic band structures for (5 × 5) p-PdSe_2_ NT under uniaxial strain; Figure S2: Electronic band structures for (9 × 9) p-PdSe_2_ NT under uniaxial strain; Figure S3: DOS and PDOS for (5 × 5) p-PdSe_2_ NT under uniaxial compressive strain; Figure S4: DOS and PDOS for (5 × 5) p-PdSe_2_ NT under uniaxial tensile strain; Figure S5: FET model based on p-PdSe_2_ nanotube with inside nanotubes air core and with dielectric core.
