# Supplementary material for: Insights into Structural, Electronic, and Transport Properties of Pentagonal PdSe2 Nanotubes Using First-Principles Calculations
Source: Nanomaterials (Basel). 2023 May 25;13(11):1728. doi: 10.3390/nano13111728 (PMC10254547; doi:10.3390/nano13111728)
Supplement: Supplementary file 1 [file nanomaterials-13-01728-s001.zip › nanomaterials-2357577-supplementary.pdf]

nanomaterials

Supplementary Materials

Insights on Structural, Electronic, and Transport Properties of the Pentagonal $PdSe_2$ Nanotubes by First-Principles Calculations

Nguyen Thanh Tien [1,*], Pham Thi Bich Thao [1], Nguyen Hai Dang [1,2], Nguyen Duy Khanh [3] and Vo Khuong Dien [4]

[1] College of Natural Sciences, Can Tho University, Can Tho 90000, Vietnam; ptbthao@ctu.edu.vn (P.T.B.T.); nhdang@nctu.edu.vn (N.H.D.)
[2] Faculty of Fundamental Science, Nam Can Tho University, Can Tho 90000, Vietnam
[3] High-Performance Computing Laboratory (HPC Lab), Information Technology Center, Thu Dau Mot University, Binh Duong 75100, Vietnam; khanhnd@tdmu.edu.vn
[4] Department of Physics, National Cheng Kung University, Tainan 701, Taiwan; vokhuongdien@gmail.com
* Correspondence: nttien@ctu.edu.vn

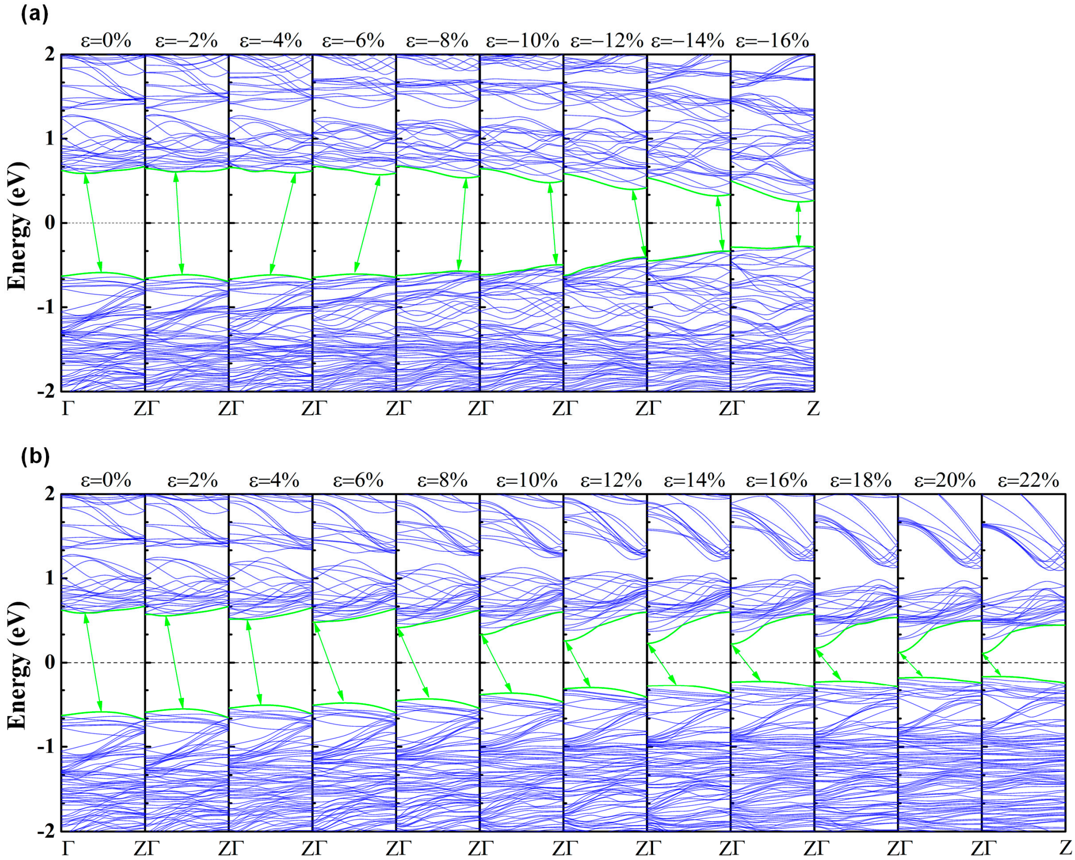

Figure S1. Electronic band structures for (5x5) p-PdSe2 NT under uniaxial compressive strain (**a**), uniaxial tensile strain (**b**). The dashed lines indicate the Fermi level.

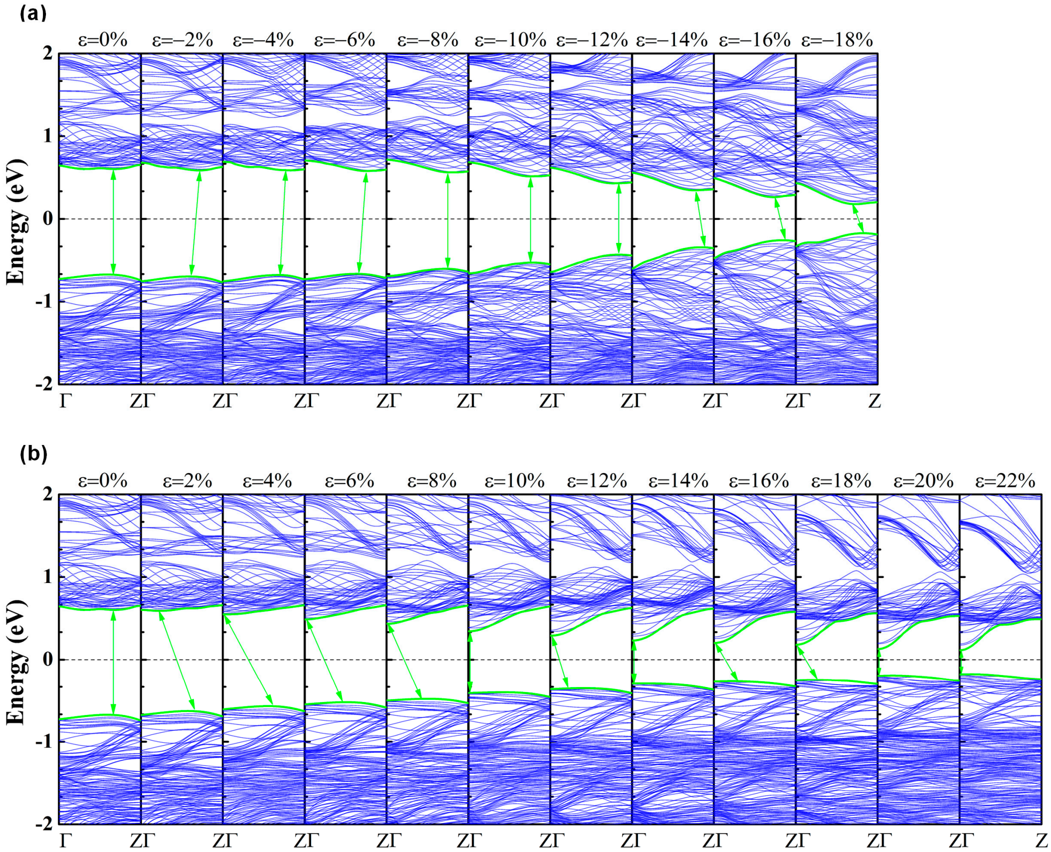

Figure S2. Electronic band structures for (9x9) p-PdSe2 NT under uniaxial compressive strain (**a**), uniaxial tensile strain (**b**). The dashed lines indicate the Fermi level.

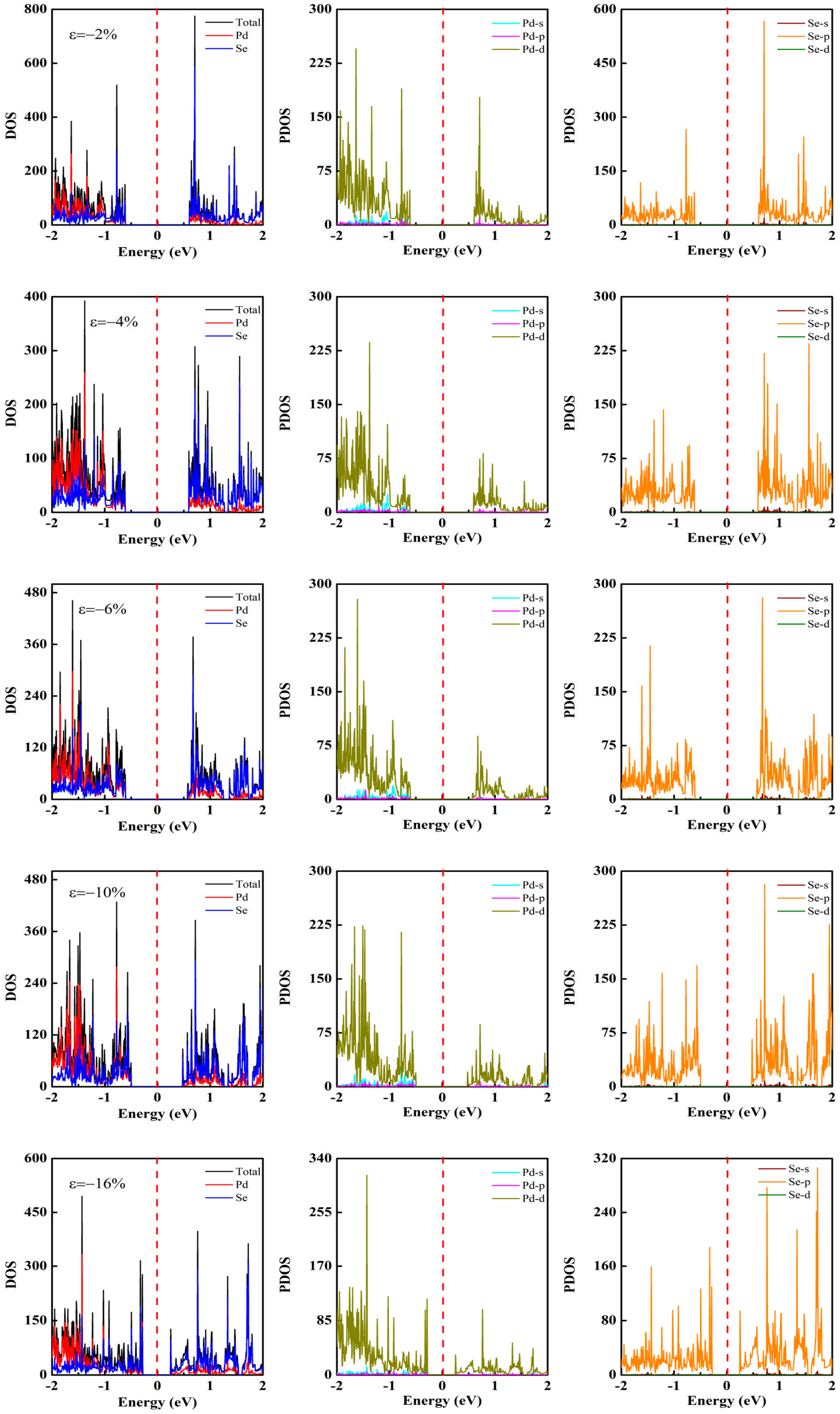

Figure S3. DOS and PDOS for (5x5) p-$PdSe_2$ NT under uniaxial compressive strain. The dashed lines indicate the Fermi level.

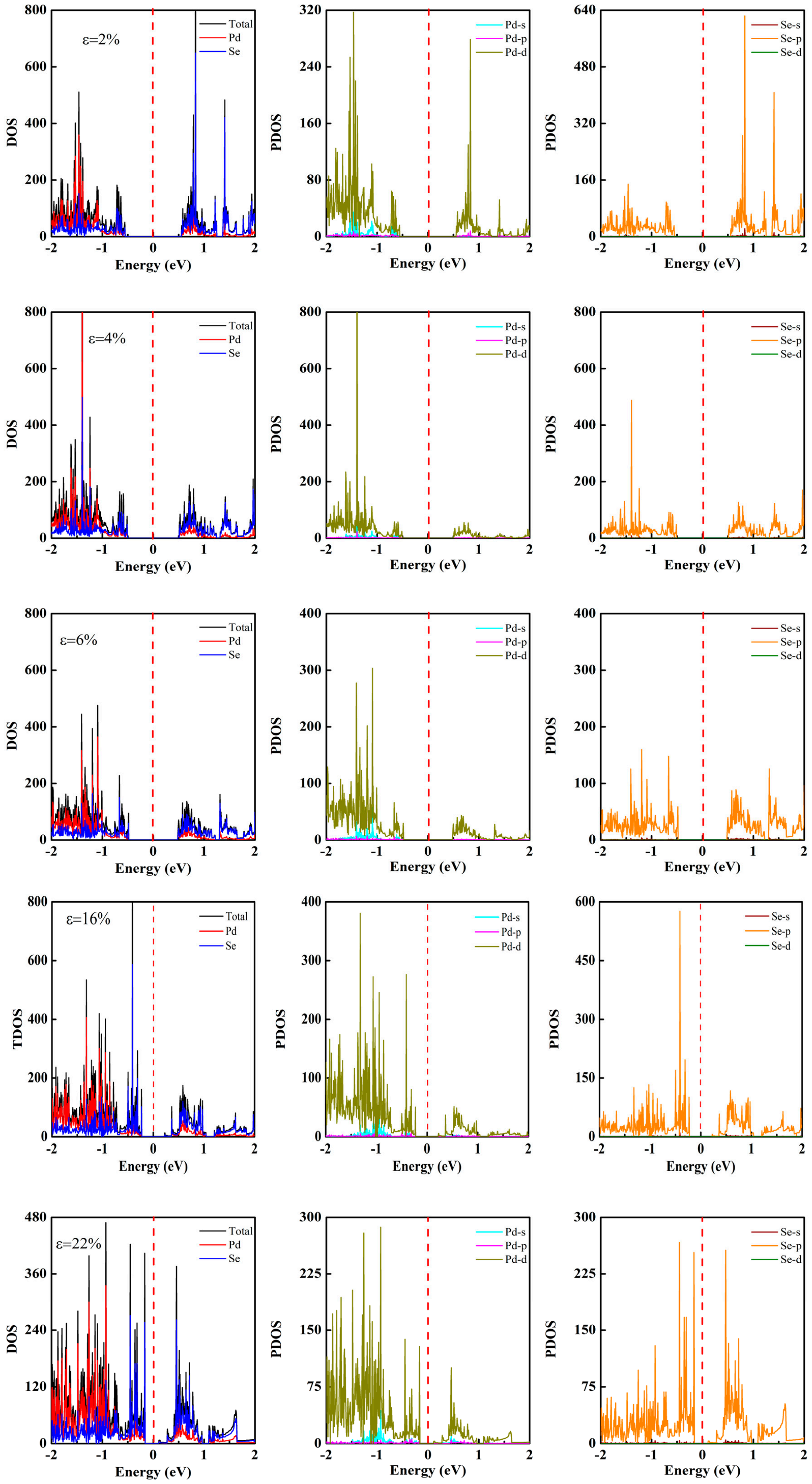

Figure S4. DOS and PDOS for (5x5) p-PdSe2 NT under uniaxial tensile strain. The dashed lines indicate the Fermi level.

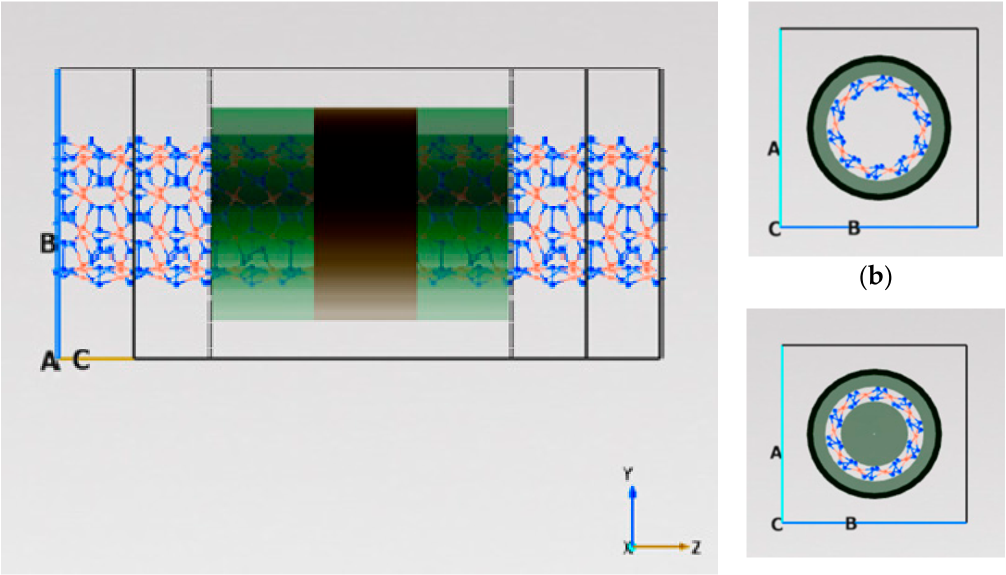

(**a**) (**b**) (**c**)

Figure S5. (**a**) FET model based on p-$PdSe_2$ nanotube. Cross-section of nanotubes without dielectric core (**b**) and with dielectric core (**c**).
